# The roles of ncRNAs in the diagnosis, prognosis and clinicopathological features of breast cancer: a systematic review and meta-analysis

**DOI:** 10.18632/oncotarget.20149

**Published:** 2017-08-10

**Authors:** Shihui Tang, Wei Fan, Jiang Xie, Qiaoling Deng, Ping Wang, June Wang, Peipei Xu, Zheng Zhang, Yirong Li, Mingxia Yu

**Affiliations:** ^1^ Department of Clinical Laboratory & Center for Gene Diagnosis, Zhongnan Hospital of Wuhan University, Wuhan, Hubei, 430071, China; ^2^ Department of Pathology, Zhongnan Hospital of Wuhan University, Wuhan, Hubei, 430071, China; ^3^ Department of Otorhinolaryngology and Head & Neck Surgery, Hunan Children's Hospital, University of South China Hengyang, Hunan, 421000, China

**Keywords:** noncoding RNA, breast cancer, meta-analysis, prognosis, diagnosis

## Abstract

**Background:**

A number of studies have shown that noncoding RNAs (ncRNAs) are abnormally expressed in breast cancers. However, the roles of ncRNAs remain unclear in breast cancer. Here, we aim to investigate the potential diagnostic and prognostic roles of ncRNAs in breast cancer.

**Methods:**

Comprehensive literature search in Medline and Web of Science and a meta-analysis were performed to identify the association between ncRNAs and diagnosis, prognosis, and clinicopathological features of breast cancer.

**Results:**

A total of 103 eligible studies, involving16, 828 independent participants, were included in the meta-analysis. In total, there were 98 individual and 11 grouped ncRNAs. 51 studies were eligible for survival analysis, 27 studies were eligible for diagnostic analysis, and 46 studies were eligible for clinicopathological features analysis. The abnormal expression of ncRNAs is associated with OS, RFS and PFS in breast cancer patients. For the diagnosis value of ncRNAs, the pooled OR and 95% CI for sensitivity, specificity, DOR and AUC on all ncRNAs were 0.83 [95% CI: 0.82- 0.84], 0.80 [95% CI: 0.79- 0.82], 24.77 [95% CI: 17.44- 35.16] and 0.9037, respectively. The analysis showed that downregulation of ncRNAs in breast cancer was associated with decreased risk of LNM, increased tumor size and PR expression, whereas, upregulation of ncRNAs was associated with increased HER2 expression.

**Conclusions:**

High expression of ncRNAs was associated with poor OS, RFS, and PFS, while low expression of ncRNAs was related to favorable OS and RFS. Meanwhile, ncRNAs have potential diagnostic value for breast cancer.

## INTRODUCTION

Breast cancer is the leading cause of cancer death in women. Early diagnosis and treatment are crucial to improve survival rate and quality of life of breast cancer patients [1]. However, because the primary breast cancers often lack typical clinical manifestations, many patients have been in advanced stage at the time of diagnosis [2]. Therefore, it is very important to find good breast cancer biological markers.

NcRNAs regulate cell differentiation, polarity, and epithelial to mesenchymal transition in breast cancer [3]. In recent years, it is known that ncRNAs served as prognosis factors in breast cancer [1, 4]. For example, Yu et al. found that down-regulation of miR-129-5p induced EMT in breast cancer cells and is associated with poor prognosis [5]. However, whether or not and how ncRNAs could be used in diagnosing and predicting prognosis of breast cancer patients remain to be determined. Systematic review and meta-analysis of data from individual studies can help to evaluate the potential clinical value of ncRNAs. This study aimed to assess the association between ncRNAs (miRNAs and long noncoding RNAs (lncRNAs)) and prognosis, diagnosis, and clinicopathological features of breast cancer.

## RESULTS

### Description of studies

**Figure 1:**
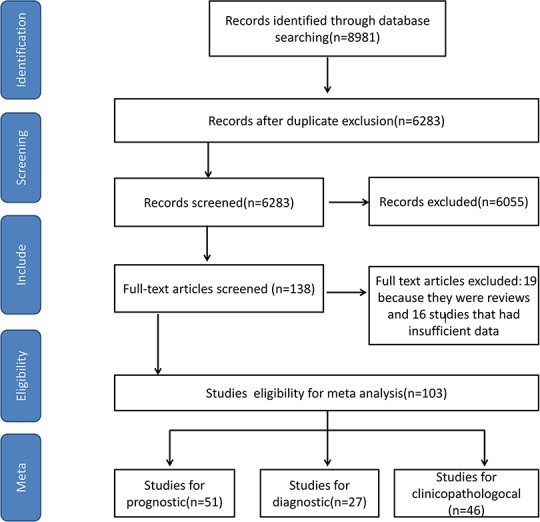
The PRISMA flow chart for selecting studies in Systematic Review and Meta-analysis

The PRISMA flow chart (Figure 1) summaries the selected studies in the review. We followed the PRISMA writing specification [6]. Our search yielded a total of 8981 reports. This was reduced to 6283 after removal of 2698 duplicates. 6055 were excluded after screening the titles and abstracts. Full-text articles were obtained for 138 studies, of which 103 were eligible for meta-analysis. 51 studies included survival data, 27 studies included diagnostic data, and 46 studies included clinicopathological features which included age, lymph node metastasis, tumor size, ER, PR, HER2, and menopausal. Low expression of ncRNAs was found in 30 studies while high expressions of ncRNAs were found in 64 studies. 32 articles investigated lncRNA, and 71 articles studied microRNA.

Micro-21 is the most commonly studied ncRNA [7–17], and all of these studies investigated its diagnostic and prognostic values in breast cancer. However, other ncRNAs showed opposite role. Madhavan [18] deemed the upregulation of miR-200b and miR-22 as unfavorable prognostic factors, while both Yao [19] and Chen [20] demonstrated that these two had potential favorable effect on breast cancer. Besides, Lu [21] proposed that breast cancer patients with high expression of HOX transcript antisense RNA (HOTAIR) had lower risks of relapse and mortality than those with lower expression. On the contrary, Gupta [22] considered that HOTAIR could reprogram chromatin state to promote breast cancer metastasis and death.

### Prognosis

51 studies reporting survival analysis were eligible for the meta-analyses (Supplementary Table 1). 44 studies reported overall survival (OS), 9 studies reported recurrence-free survival (RFS), and 4 studies reported progression/event/disease-free survival (PFS) (Table 1). Summarized hazard ratio (HR) was used as effect size to estimate the relationship between expression of ncRNAs and breast cancer survival. Random-effects model was used for the pooled analysis to detect heterogeneity. In the 44 studies reporting OS, high expression of 19 ncRNAs was associated with improved breast cancer survival rates (HR=0.33, 95% confidence interval (95% CI) : 0.23-0.47) (Supplementary Figure 1B) and 21 ncRNAs were associated with an increased risk of death (HR=2.63, 95% CI: 2.27-3.05) (Supplementary Figure 1A).

**Table 1:** Summary HR of ncRNAs for breast cancer

Survival analysis		No. of studies	No. of patients	Pooled HR	Heterogeneity
OS	Down	19	4472	0.33[0.23-0.47]	79%
	Up	25	6854	2.63[2.27-3.05]	53%
RFS	Down	4	2605	0.68[0.53,0.87]	67%
	Up	5	7447	2.70[1.91,3.81]	0%
PFS	Down	1	57	0.40[0.17,0.94]	/
	Up	3	442	2.09[1.41,3.11]	64%

Seven ncRNAs were investigated in more than two studies: miR-21 (n=8 studies), miR-200a (n=2 studies), miR-200b (n=2 studies), miR-200c (n=2 studies), miR-22 (n=2 studies), miR-124 (n=2 studies), miR-210 (n=2 studies), and HOTAIR (n=2 studies). By combining these studies, the pooled HR and 95% CI were as follows: miR-21 (HR=1.97, 95% CI: 1.55-2.44); miR-200a (HR=3.24, 95% CI: 1.30-8.07); miR-200b (HR=2.08, 95% CI: 0.54-8.01); miR-200c (HR=3.41, 95% CI: 1.91-6.09); miR-22 (HR=0.88, 95% CI: 0.36-2.19); miR-124 (HR=0.71, 95% CI: 0.55-0.92); miR-210 (HR=0.71, 95% CI: 0.55-0.92); HOTAIR (HR=1.21, 95% CI:0.16-8.93) (Supplementary Figure 3).

Similarly, 9 studies were eligible for the RFS, with 4 down-regulated ncRNAs (HR=0.71, 95% CI: 0.55-0.92) and 5 up-regulated ncRNAs (HR=2.70, 95% CI: 1.91-3.81) were associated with risk of recurrence (Supplementary Figure 1D). After combining two studies, the result for MALAT1 showed a pooled HR of 2.36 (95% CI: 1.55-3.60) in terms of RFS. For the PFS, three studies addressed 4 up-regulated ncRNAs (HR=2.09, 95% CI: 1.41-3.11) and one down regulated (Supplementary Figure 1C).

### Diagnosis

Analysis of data from diagnostic accuracy studies including 27 original studies reported 1 lncRNA and 47 miRNAs (Supplementary Table 2). 13 ncRNAs are down regulated and the others are up regualted. All articles including 2287 patients and 1644 healthy control were published between 2010 and 2016. Significant heterogeneity was observed among the 27 studies in sensitivity and specificity analyses (I^2^=86.1% and 93.8%, respectively). The pooled estimates for sensitivity, specificity, positive likelihood ratios (PLR), negative likelihood ratios (NLR), diagnostic odds ratios (DOR) and Area Under Curve (AUC) of all ncRNAs were 0.83 [95% CI: 0.82-0.84], 0.80 [95% CI: 0.79-0.82], 4.51 [95% CI: 3.62-5.62], 0.21 [95% CI: 0.17-0.25], 24.77 [95% CI: 17.44-35.164] and 0.9037, respectively.

The most commonly studied ncRNA is miR-21 (n=9 studies). Pooled estimates of sensitivity, specificity, PLR, NLR, DOR and AUC associated with miR-21 were 0.77 [95% CI: 0.74-0.82], 0.84 [95% CI: 0.78- 0.88], 4.25 [95% CI: 2.40- 7.52], 0.23 [95% CI: 0.12- 0.46], 18.141 and 0.8982 (Supplementary Figure 4; Supplementary Figure 5; Supplementary Figure 6).

### Clinicopathological features

**Figure 2:**
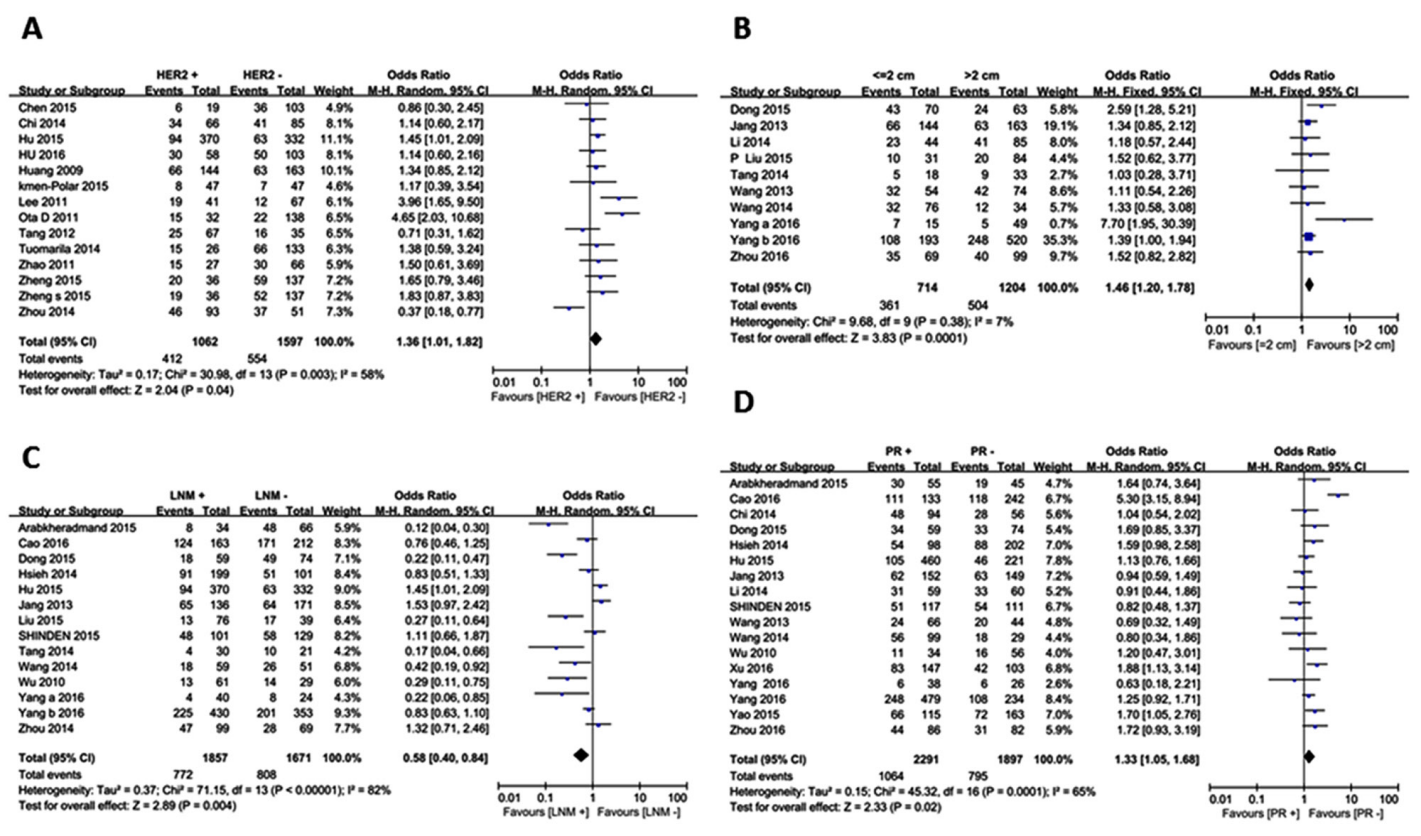
Forest plots for Clinicopathological features Up-regulated ncRNAs : **(A)** Her2; down-regulated ncRNAs: **(B)** Tumor size; **(C)** Lymph node metastasis; **(D)** PR.

46 studies including 24 microRNAs and 15 lncRNAs were included in analyses of the relationship between ncRNA and clinicopathological features (Supplementary Table 3), such as age, lymph node metastasis (LNM), tumor size, estrogen receptor (ER), progesterone receptor (PR), human epidermalgrowth factor receptor-2 (HER2), and menopausal factors (Table 2). 24 ncRNAs were up-regulated and 15 ncRNAs were down-regulated in breast cancer. The results suggested that upregulation of ncRNAs was positively correlated with the expression of HER2 (odd ratio (OR)=1.36, 95% CI: 1.10- 1.82). Meanwhile, an inverse correlation between down-regulated ncRNAs and LNM (OR=0.53, 95% CI: 0.36- 0.78), positive correlation between down-regulated ncRNAs and tumor size (OR=1.47, 95% CI: 1.19- 1.82) and the expression of PR (OR=1.33, 95% CI: 1.05- 1.68) was noted (Figure 2). There were no other statistically significant associations between ncRNA and other factors (Supplementary Figure 7–Supplementary Figure 14).

**Table 2:** Summary diagnostic accuracy of ncRNAs for breast cancer

Analysis	No.	Pooled Sen	Pooled Spe	PLR	NLR	AUC
Sum	27	0.83(0.82- 0.84)	0.80(0.79- 0.82)	4.51(3.62- 5.62)	0.21(0.17- 0.25)	0.9037
ncRNA profiles						
combine	7	0.86(0.84- 0.89)	0.87(0.84- 0.89)	7.22(4.26- 12.23)	0.16(0.11- 0.24)	0.9240
single	24	0.82(0.80- 0.83)	0.79(0.77- 0.80)	4.16(3.28- 5.26)	0.22(0.18- 0.27)	0.8951
Sample types						
tissue	7	0.81(0.79- 0.83)	0.77(0.74- 0.80)	3.75(2.48- 5.67)	0.26(0.18- 0.39)	0.8744
blood	22	0.83(0.82- 0.84)	0.82(0.80- 0.84)	4.93(3.838- 6.34)	0.18(0.15- 0.23)	0.9147

Sen: sensitivity, Spe: specificity, PLR: Positive likelihood ratio, NLR: negative likelihood ratio, AUC: area under concentration-time curve

### Subgroup analyses and meta-regression

#### Prognosis

**Figure 3:**
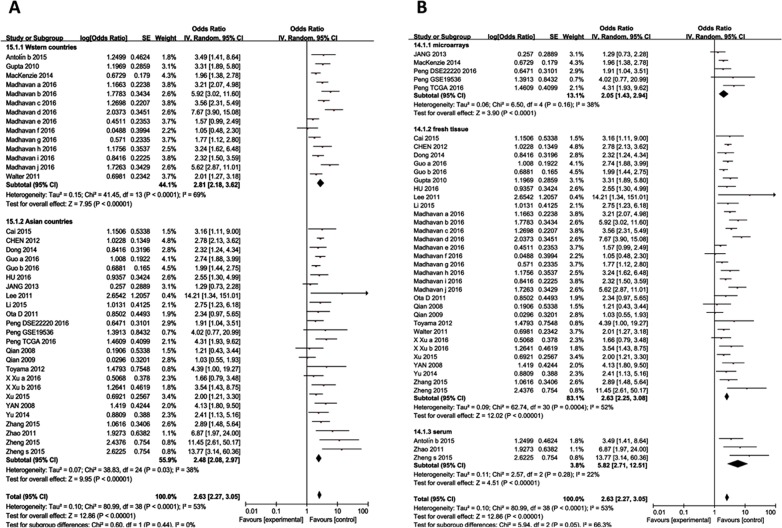
Subgroup analyses for OS of breast cancer patients **(A)** Subgroup analyses based on races for up-regulated ncRNAs; **(B)** Subgroup analyses based on sample types for up-regulated ncRNAs.

We performed meta-regression analysis but didn't found the publication year and RNA type which can explain the heterogeneity in up regulation ncRNAs [Chi^2^=79.90, df=36 (P<0.0001); I^2^=55%]. Then, we performed subgroup analysis based on races and sample type. There was no significant heterogeneity in the tissue microarrays subgroup [Chi^2^=2.60, df =4 (P=0.63); I^2^=0%] and blood subgroup [Chi^2^=2.57, df=7 (P=0.28); I^2^=22%]. But significant heterogeneity [Chi^2^=62.74, df=30 (P=0.0004); I^2^=52%] was observed in fresh tissue subgroup (Figure 3B).

#### Diagnosis

**Figure 4:**
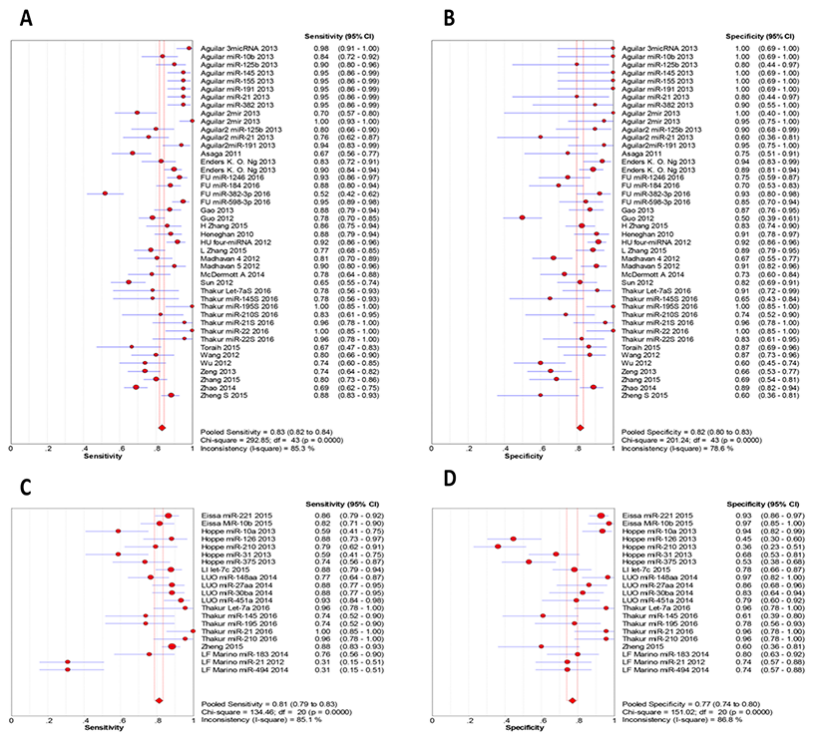
Sensitivity and specificity in subgroup analysis based on sample type **(A)** the pooled sensitivity for blood; **(B)** the pooled specificity for blood; **(C)** the pooled sensitivity for tissues; **(D)** the pooled specificity for tissues.

**Figure 5:**
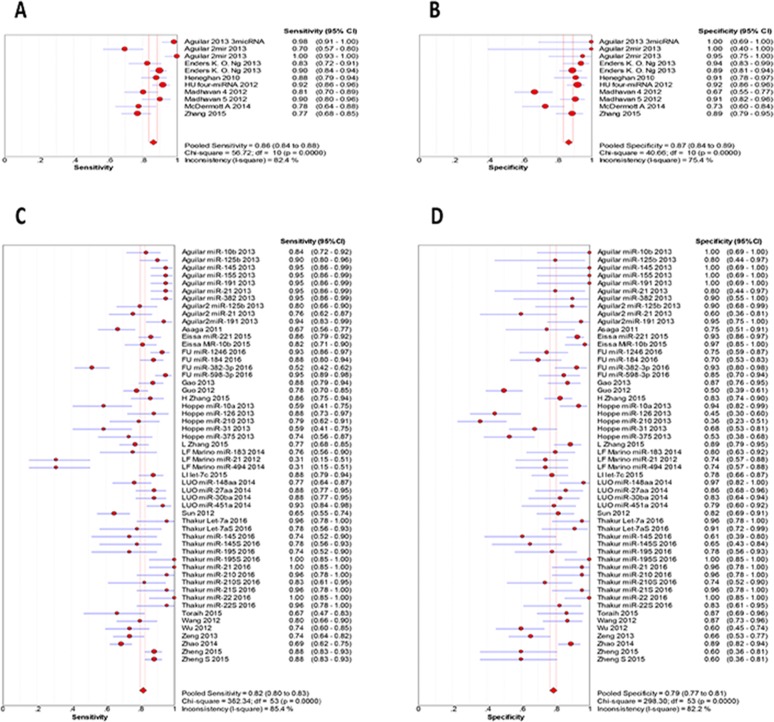
Sensitivity and specificity in subgroup analysis based on constituent **(A)** the pooled sensitivity for single ncRNA; **(B)** the pooled specificity for single ncRNA; **(C)** the pooled sensitivity for multiple ncRNAs; **(D)** the pooled specificity for multiple ncRNAs.

To study the diagnostic value, we performed subgroup analysis based on sample type. We found that the ncRNAs extracted from blood have higher sensitivity, specificity and AUC than those from tissue (Figure 4). Furthermore, diagnosis based on multiple ncRNAs showed higher accuracy than signle ncRNA (Table 3, and Figure 5). This information revealed a high potential diagnostic value of multiple ncRNAs from blood for breast cancer detection.

**Table 3:** Relationship between clinicopathological features and ncRNAs in breast cancer

Survival analysis		No. of studies	No. of patients/controls	Pooled HR	Heterogeneity
Age	Down	10	881/788	1.05[0.80-1.38]	37%
	Up	15	934/1022	1.01[0.84-1.22]	0%
LNM	Down	13	1721/1500	0.58[0.40,0.84]	81%
	Up	17	1480/1441	1.00[0.73,1.36]	70%
Tumor Size	Down	9	714/1204	1.47[1.19,1.82]	7%
	Up	18	1265/1596	0.80[0.60,1.05]	65%
ER	Down	15	2499/1535	1.25[0.98,1.61]	66%
	Up	25	2133/1818	0.91[0.73,1.14]	59%
PR	Down	16	2291/1897	1.33[1.05,1.68]	65%
	Up	23	1964/1981	1.15[0.94,1.41]	50%
HER2	Down	11	849/1389	0.68[0.42,1.11]	85%
	Up	14	1062/1597	1.36[1.10,1.82]	58%
Menopausal	Down	2	166/159	1.11[0.72,1.69]	0%
	Up	11	866/917	1.13[0.93,1.36]	15%

### Influence analysis and publication bias

**Figure 6:**
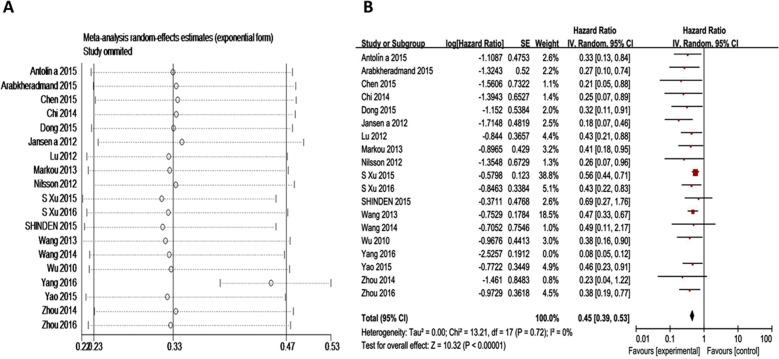
Subgroup analyses for OS of breast cancer patients **(A)** Influence analysis for down-regulated ncRNAs; **(B)** Forest plots for down-regulated ncRNAs after excluding the outlier.

In influence analysis for studies on highly expressed ncRNA, pooled results were not substantially altered after removing any one of the studies, suggesting that the pooled result is stable. In contrast, in down regulated part, we found an outlier and once this outlier was excluded [23], I^2^ went down from 79% to 0% [Chi^2^=13.21, df =17 (P=0.72); I^2^=0%] (Figure 6B).

Risk of publication bias is a significant concern in prognostic studies [24]. Begg's funnel plot and Egger's test were used to evaluate the publication bias in the published literature [25]. For OS, Begg's funnel plot (P=0.045) and Egger's test (P=0.053) showed certain publication bias (Supplementary Figure 1; Supplementary Figure 2A). By using trim-and-fill Method, a symmetrical funnel plot was produced. Hypothetical negative unpublished studies imputed to mirror the positive studies that cause funnel plot asymmetry (Figure 3B) [26]. The pooled analysis showed a negative relationship between high expression ncRNA and OS (HR, 0.82 [95% CI, 0.65-0.99]), and the result has good stability (Supplementary Figure 1B). For low expression ncRNA, the result of Begg's funnel plot is opposite to Egger's test. After excluded the outlier as mentioned above, funnel plot transformed to symmetry.

The Deek's funnel plot asymmetry test was used to evaluate the publication bias in diagnostic analysis [25]. The result (P=0.12) confirmed that there was no significant publication bias (Supplementary Figure 6).

Influence analysis and publication bias examination were implemented for every clinicopathological feature, in highly and lowly expressed ncRNAs, respectively. P-values from Begg's funnel plot and Egger's test were all larger than 0.05. Since the studies of down-regulated ncRNAs were less than twenty, we performed the Begg's funnel plot and founded the funnel is asymmetric. Meanwhile, influence analysis results manifested the stability of results. In the high expression series, only HER2 group existed significant publication bias.

## DISCUSSION

According to the GLOBOCAN estimates, about 14.1 million new cancer cases and 8.2 million deaths occurred in 2012 worldwide [27]. Human breast carcinoma is one of the most frequent cancers in the female and deeply threatens woman health and life quality. In this review, we gathered published papers to study the relationship between ncRNAs and prognosis, diagnosis, and clinicopathological features in breast cancer.

By systematically analyzing published original studies, we found that ncRNA have predictive value for prognosis in breast cancer. Combined HRs suggest that ncRNA are independent risk factors for OS in breast cancer patients. In addition, ncRNAs appear to be independent prognostic risk factors for RFS and PFS in breast cancer patients. However, these analyses had significant heterogeneity in terms of sample type and multivariable models. Large sample microarrays subgroup showed no significant heterogeneity but the effect size (HR=1.83) was weaker than others, indicating the possibility of overestimating effect size. Besides, our finding also suggest that some ncRNAs alone (miR-124, miR-210, miR-21, miR-200a, miR-200c) are independent risk factors for OS in breast cancer.

The detection methods for breast cancer such as imaging examination and pathological examination had certain limitations, including radiation, invasion and low diagnostic accuracy. Noninvasive biomarkers, such as CEA and CA15-3, are widely used in clinic. However, these markers have low sensitivity and specificity for breast screening [9, 28]. Zeng et al [29] have demonstrated that these markers may be more suitable for advanced breast cancer. Thus, it is important to identify effective and noninvasive tumor biomarkers for early detection and diagnosis. In this meta-analysis, the overall AUC of SROC is 0.9037, indicating a high accuracy of ncRNAs. The DOR value also evaluates the accuracy of a diagnostic test, with higher values indicating better performance[30]. DOR value less than 1.0 does not approve its competent role as a biomarker in the diagnostic test [31]. Nevertheless, the pooled DOR in this work reached up to 24.767, suggesting ncRNA can be used as a non-invasive indicator for breast cancer diagnosis. Besides, the sensitivity and specificity of multiple ncRNAs [18, 32–37] are higher than the single ones, suggesting combined ncRNAs could be useful for the diagnosis of breast cancer.

The threshold effect is one of the leading causes of heterogeneity in a diagnostic meta-analysis [38], and mainly caused by the different cut-off values used in individual studies [39]. Heterogeneity generated by non-threshold effect is usually addressed via pooled DOR [30]. The P values in Cochran's Q test were all less than 0.01 in our study, accompanied by I^2^ more than 50 %, suggesting that heterogeneity in the overall and subgroup analyses was attributed to threshold effect and non- threshold effect. Deek's funnel plot asymmetry test indicated no significant publication bias in the diagnostic analysis.

In the clinicopathological features part, down-regulated ncRNAs were negatively related to tumor size and LNM, but positively related to the expression of PR; and up-regulated ncRNAs were positively related to the expression of HER2. However, the result of the relationship between down-regulated ncRNAs and HER2 exist significant heterogeneity, and after excluding the article [19] that stood out in the influence analysis, heterogeneity no longer has significance (Supplementary Figure 10). The LNM group showed large heterogeneity as well, and subgroup analyses by ncRNA type and sample type failed to locate the source of heterogeneity. Influence analyses for other aspects showed fine stability.

Three ncRNAs (miR-2 (n=6), MALAT 1 (n=4), miR-124 (n=2)) were studied in more than two studies. Our comprehensively analyzed data suggested that the expression of miR-21 was positively related to HER2 (OR=2.74, 95% CI: 1.13-6.62), negatively related to ER (OR=0.57, 95% CI: 0.34-0.96) and positively related to age (OR=0.57, 95% CI: 0.35-0.95). Meanwhile, the expression of MALAT1 was negatively related to tumor size (OR=1.29, 95% CI: 1.05-1.59). The expression of mir-124 was negatively related to LNM (OR=0.17, 95% CI: 0.10-0.31).

Most of the meta-analysis only evaluated the relationship between a type of ncRNA and many cancers. In our study, data were pooled rationally. We have analyzed the role of all ncRNAs in breast cancer including prognosis, diagnosis, and clinicopathological features. Besides, single ncRNA was also estimated. In diagnosis, we analyzed single ncRNA and multiple ncRNAs, respectively. The results suggested joint detection is better than single detection.

### Limitations

Most of the included ncRNAs have been studied only once previously, making it difficult to systematically evaluate the clinical value of one specific ncRNA. There are several other limitations in our study. First, this review is only based on the results of databases of published studies, and did not include study registers or gray literature. This could be the source of publication bias. Second, our meta-analysis combines multiple studies, which contains different types, samples, cutoff values, and HRs, all of which may cause statistical heterogeneity. Third, many potential biomarkers being analyzed in our study are not used in clinical practice yet.

## CONCLUSION

Taken together, our results indicated the potential value of ncRNAs in the prognosis and diagnosis of breast cancer. But considerable shortcoming might influence our final estimates. Therefore, future standardized researches with high quality are needed to verify these results.

## MATERIALS AND METHODS

### Study strategy and eligibility criteria

We conducted comprehensive literature searches in Medline and Web of Science for eligible studies up to Sep, 2016, using the keywords[(ncRNAs or microRNAs or miRNAs or long non-coding RNAs) AND (breast or breast cancer)], with publication language limited to English.

Case-control reports were identified that explored the association of any single or combination of relevant ncRNAs with one or more of the following aspects: survival, diagnosis and clinical features of breast cancer patients.

In order to be eligible for inclusion in meta-analysis, studies had to provide the effect size and CI for the association of ncRNAs with outcomes, or appropriate data for the effect size and CI could be calculated. For survival analysis, we extracted the HRs and 95% CI [40] for overall survival (OS, duration of time from day of diagnosis to the day of death due to any cause), recurrence-free survival (RFS, duration of time from day of cure from cancer to the day evidence of cancer progression/recurrence is identified), progression/event/disease-free survival (PFS, duration of time from the day of first treatment to the day evidence of cancer progression are identified or the patient dies of any cause). In diagnostic articles, the sensitivity and specificity are extracted to construct two-by-two tables. ORs and 95% CI were used to investigate the relationship between expression levels of ncRNAs and clinicopathological features. At the same time, we excluded review, letters, case reports, guidelines, and some studies without complete data.

Two reviewers independently screened titles and abstracts of all identified records according to pre-specified inclusion and exclusion criteria. Disagreements were resolved by a third reviewer. Full text articles were obtained for all included studies and were screened again for inclusion or exclusion by two reviewers independently, with disagreements resolved by discussion.

### Quality assessment

Three researchers independently reviewed and evaluated eligible studies assessed by the Newcastle-Ottawa quality assessment scale (NOS)[41, 42]. The methodological quality of diagnosis part in this study was performed with Quality Assessment of Diagnostic Accuracy Studies (QUADAS-2) criteria [41].

### Data extraction

Data were extracted from eligible articles as follow. General information: first author's name, year of publication, country of the study, type of ncRNAs, sample size; characteristics of participants: (1) HRs and 95% CI were extracted for survival effect size. Since effect estimates extracted from multivariate analysis (e.g. Cox regression) that are affected by other variables, HR and 95% CI were not directly extracted [10, 43–46]; approximations of HRs were indirectly calculated based on the correlative statistics using the methods described by Tierney et al [28]. When ncRNA expression levels were subdivided into low versus medium versus high groups, only data for the comparison between high versus low expression levels were extracted [14, 21, 47, 48]; (2) For diagnostic studies data were extracted relating to: sensitivity and specificity (two-by-two tables); (3) clinicopathological characteristics are age, tumor size, menopausal, lymph node metastasis, and the expression of growth factor receptors (estrogen receptor, ER; progesterone receptor, PR; epidermal growth factor receptor 2, HER2).

### Statistical analysis

Data syntheses were conducted using Review Manager 5.2 and Meta-Disc 1.4. Publication bias examination, and influence analyses were performed using STATA 11.0. The HR with the corresponding 95% CI for OS, RFS and PFS were calculated to evaluate the prognostic value of ncRNA [49, 50]. HR>1 imply that patients with lower expression of the ncRNA had better prognosis than patients with higher expression. The true positive (TP), false positive (FP), false negative (FN), true negative (TN) based on two-by-two tables, that were used to consider sensitivity (SE), specificity (SP), positive likelihood ratio (PLR), negative likelihood ratio (NLR), and summary receiver operator characteristic (SROC) curve. These data were used to assess the diagnosis value of ncRNAs in breast cancer. Odds ratios (ORs) and the 95% CI were calculated to analyze the relationship between ncRNA and clinicopathological features. P<0.05 was considered statistically significant. Heterogeneity was analyzed using the Q and I^2^ statistics [41, 42]. P < 0.1 indicated presence of heterogeneity. I^2^ > 50% was defined as significant heterogeneity.

## SUPPLEMENTARY MATERIALS FIGURES AND TABLES


