# Supplementary material for: The roles of ncRNAs in the diagnosis, prognosis and clinicopathological features of breast cancer: a systematic review and meta-analysis
Source: Oncotarget. 2017 Aug 10;8(46):81215–25. doi: 10.18632/oncotarget.20149 (PMC5655276; doi:10.18632/oncotarget.20149)
Supplement: Supplementary file 1 [file oncotarget-08-81215-s001.pdf]

The roles of ncRNAs in the diagnosis, prognosis and clinicopathological features of breast cancer: a systematic review and meta-analysis

SUPPLEMENTARY MATERIALS

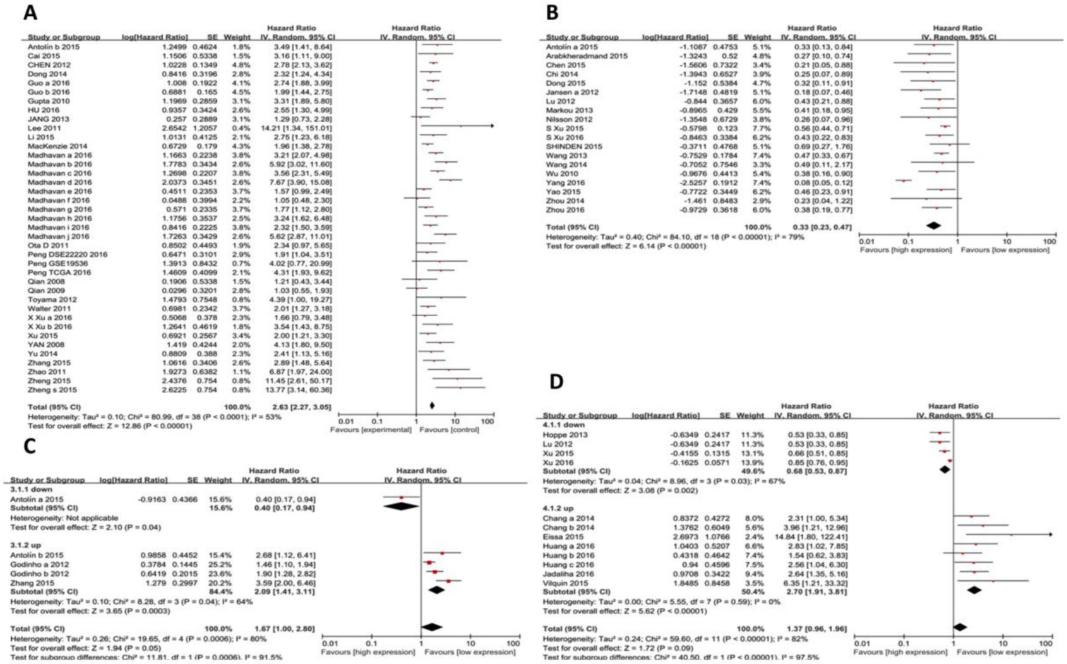

Supplementary Figure 1: Forest plots for prognostic meta-analyses. (A) up regulated ncRNAs was a risk factor for the OS of breast cancer; **(B)** the pooled HR for OS of down regulated ncRNAs; **(C)** Meta-analysis of the ncRNAs in the PFS of breast cancer; **(D)** Meta-analysis of the ncRNAs in the RFS of breast cancer.

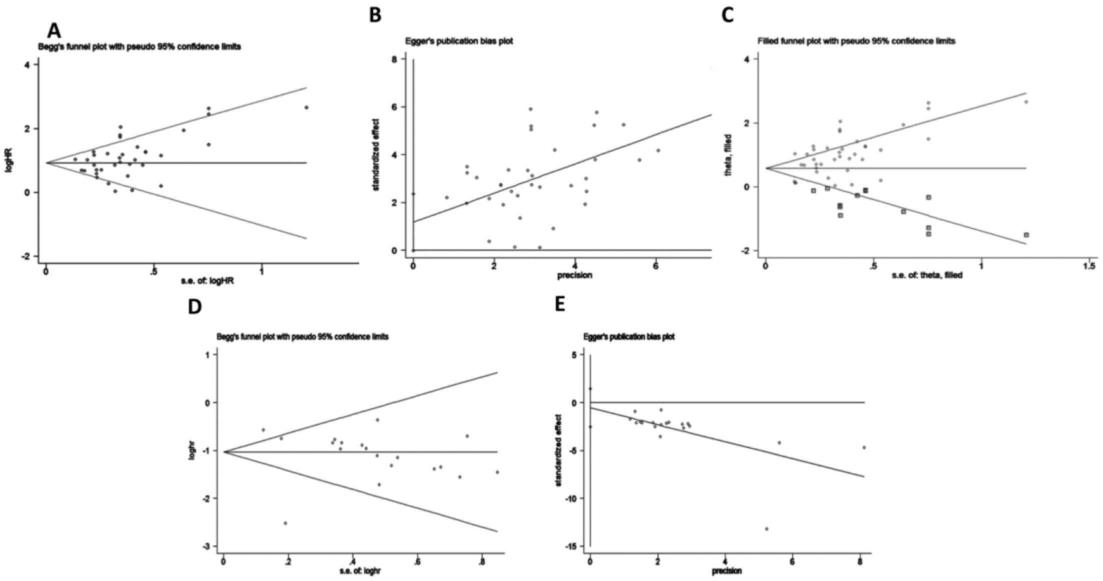

Supplementary Figure 2: Publication bias. **(A)** Begg's funnel plot for OS of up-regulated ncRNAs; **(B)** Egger's test for OS of up-regulated ncRNAs; **(C)** Filled plot for OS of up-regulated ncRNAs; **(D)** Begg's funnel plot for OS of down-regulated ncRNAs; **(E)** Egger's test for OS of down-regulated ncRNAs.

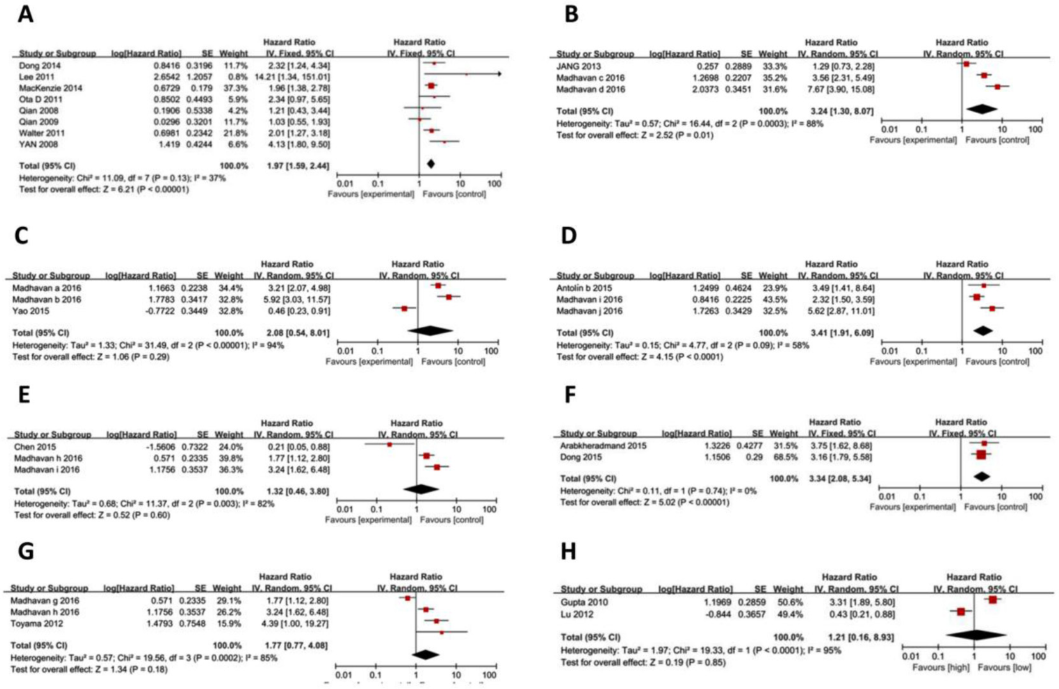

Supplementary Figure 3: Forest plots for OS. **(A)** mir21; **(B)** mir200a; **(C)** mir200b; **(D)** mir200c; **(E)** mir22; **(F)** mir124; **(G)** mir210; **(H)** HOTAIR.

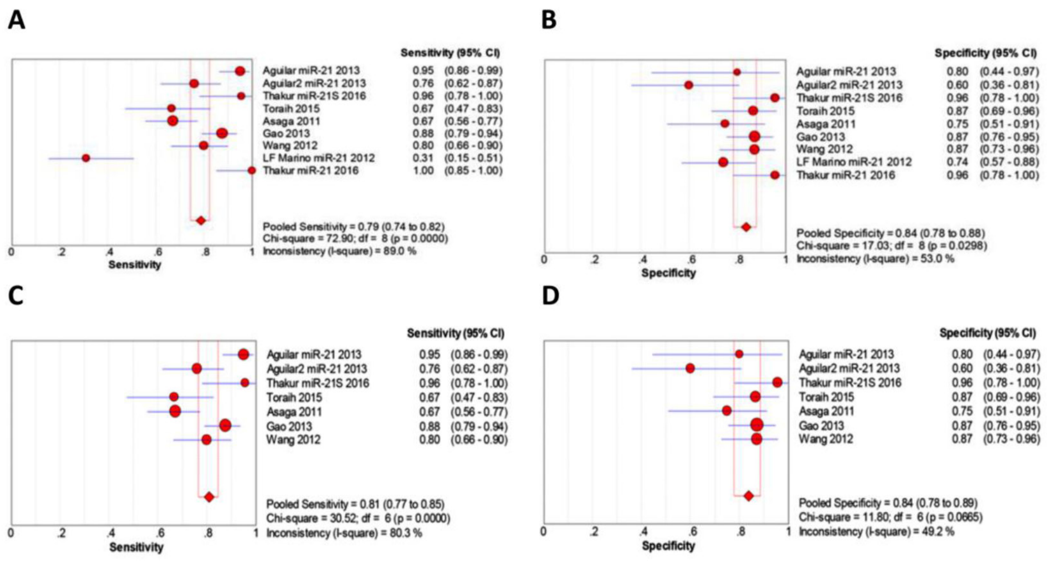

Supplementary Figure 4: Diagnostic meta-analysis of miR21. **(A)** Sensitivity of mir21; **(B)** Specificity of mir21; **(C)** Sensitivity of mir21 extracted from serum; **(D)** Specificity of mir21 extracted from serum.

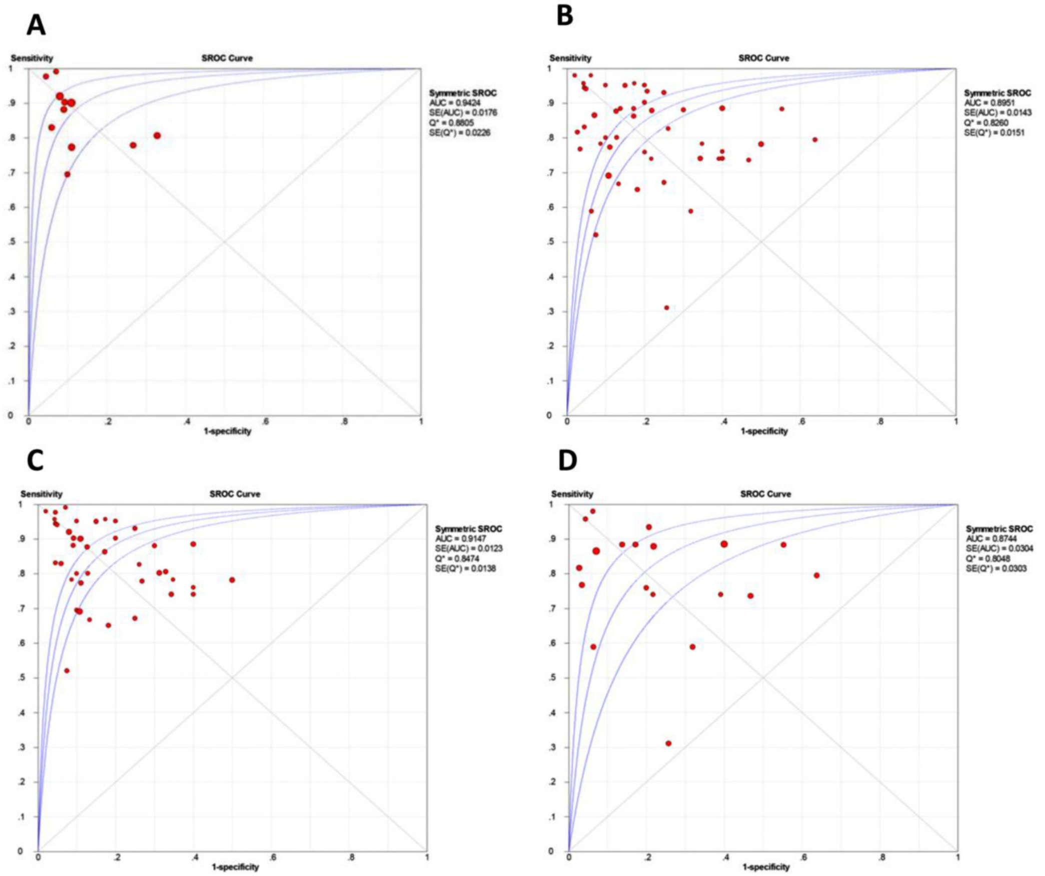

Supplementary Figure 5: The SROC curve of the subgroup analyses for the diagnosis of breast cancer. **(A)** Combine ncRNAs analysis; **(B)** Single ncRNA-based analysis; **(C)** Single ncRNA-based analysis; **(D)** Tissue-based ncRNA analysis.

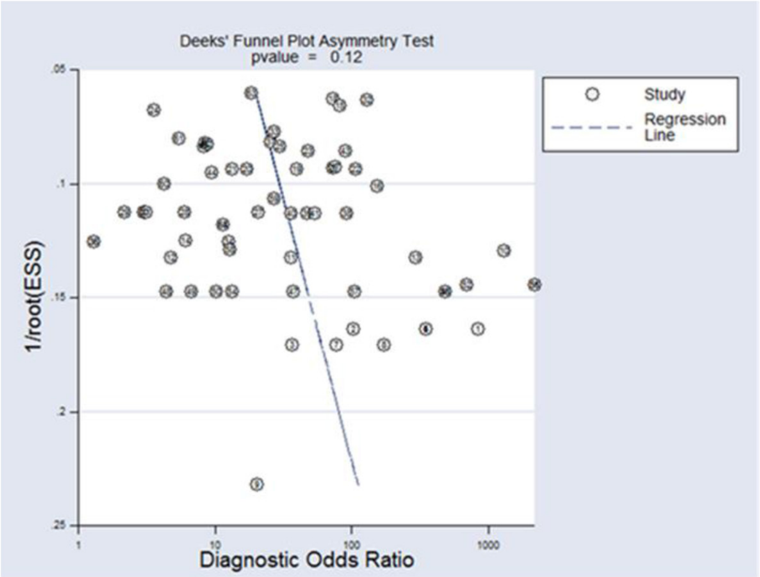

Supplementary Figure 6: Deeks' funnel plot asymmetry test for total ncRNAs assays, P=0.12.

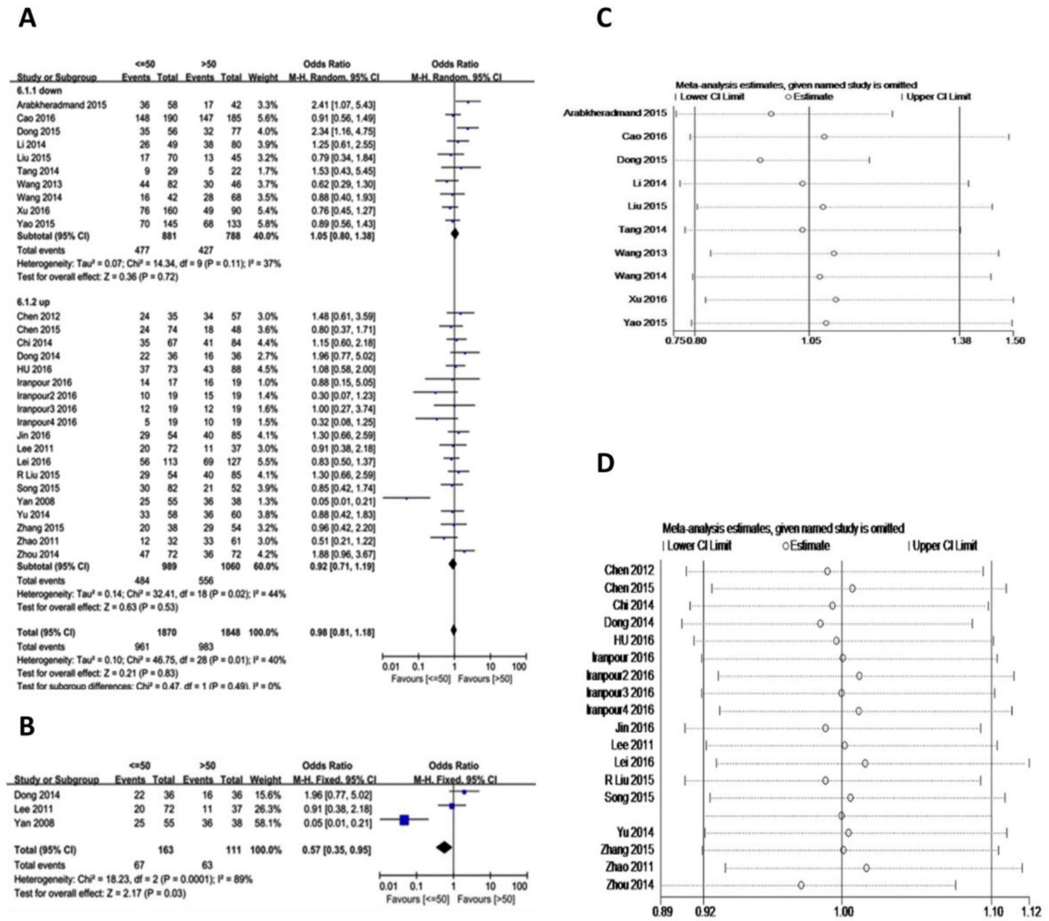

Supplementary Figure 7: Meta-analysis of age. (A) Forest plots for the relation between ncRNAs and age of breast cancer patients; **(B)** Forest plots for the relation between mir21 and age of breast cancer patients; **(C)** Influence analysis for the relation between down-regulated ncRNAs and age of breast cancer patients; **(D)** Influence analysis for the relation between up-regulated ncRNAs and age of breast cancer patients.

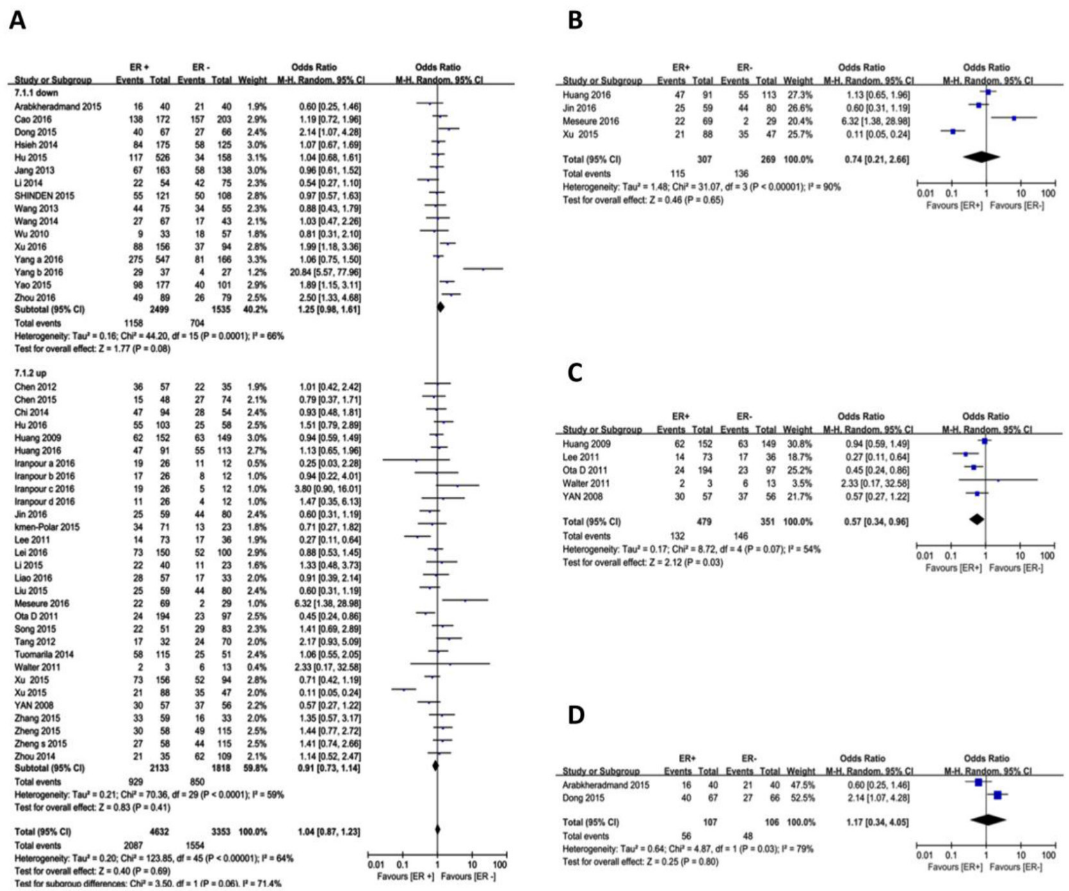

Supplementary Figure 8: Meta-analysis of ER. (A) Forest plots for the relation between ncRNAs and the expression of ER; **(B)** Forest plots for the relation between MALAT1 and the expression of ER; **(C)** Forest plots for the relation between mir21 and the expression of ER; **(D)** Forest plots for the relation between mir124 and the expression of ER.

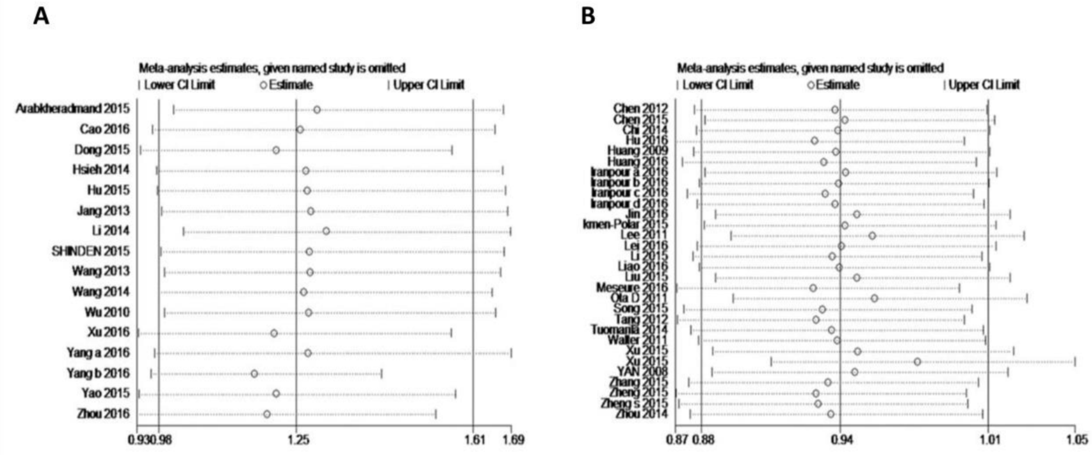

Supplementary Figure 9: Influence analysis for the relation between ncRNAs and the expression of ER. (A) down-regulated ncRNAs; **(B)** up-regulated ncRNAs.

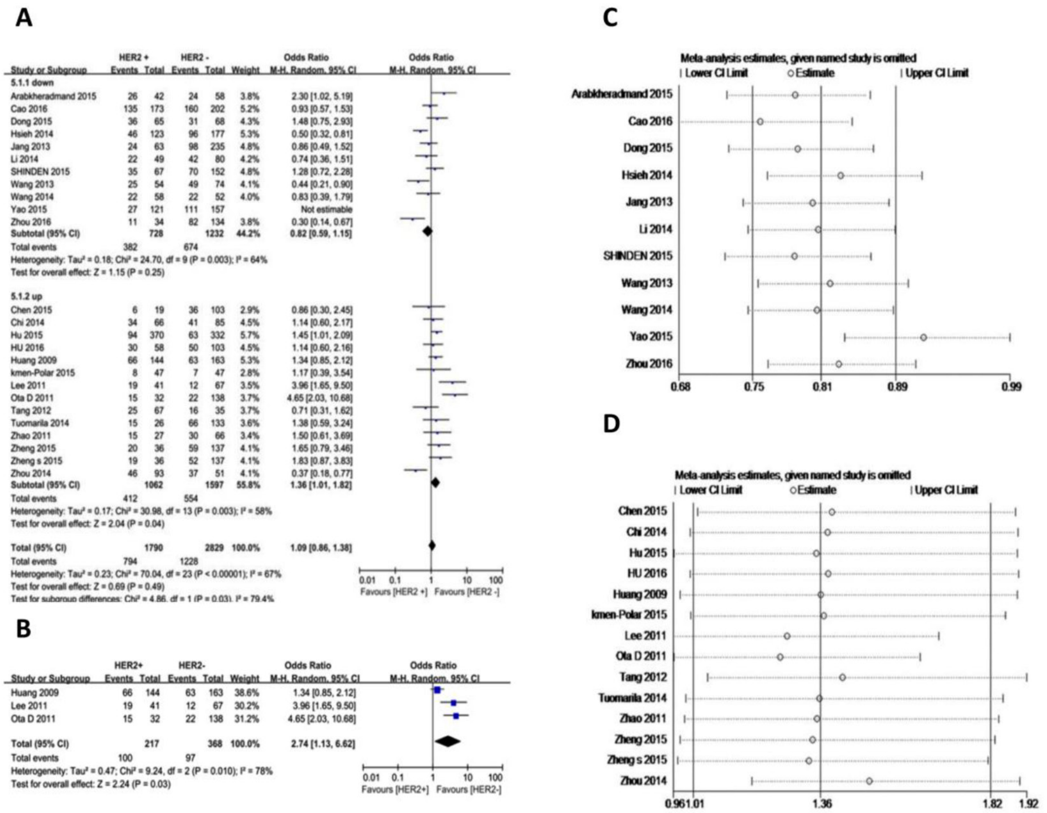

Supplementary Figure 10: Meta-analysis of HER2. A. Forest plots for the relation between ncRNAs and the expression of HER2; **(B)** Forest plots for the relation between mir21 and the expression of HER2; **(C)** Influence analysis for the relation between down-regulated ncRNAs and the expression of HER2; **(D)** Influence analysis for the relation between up-regulated ncRNAs and the expression of HER2.

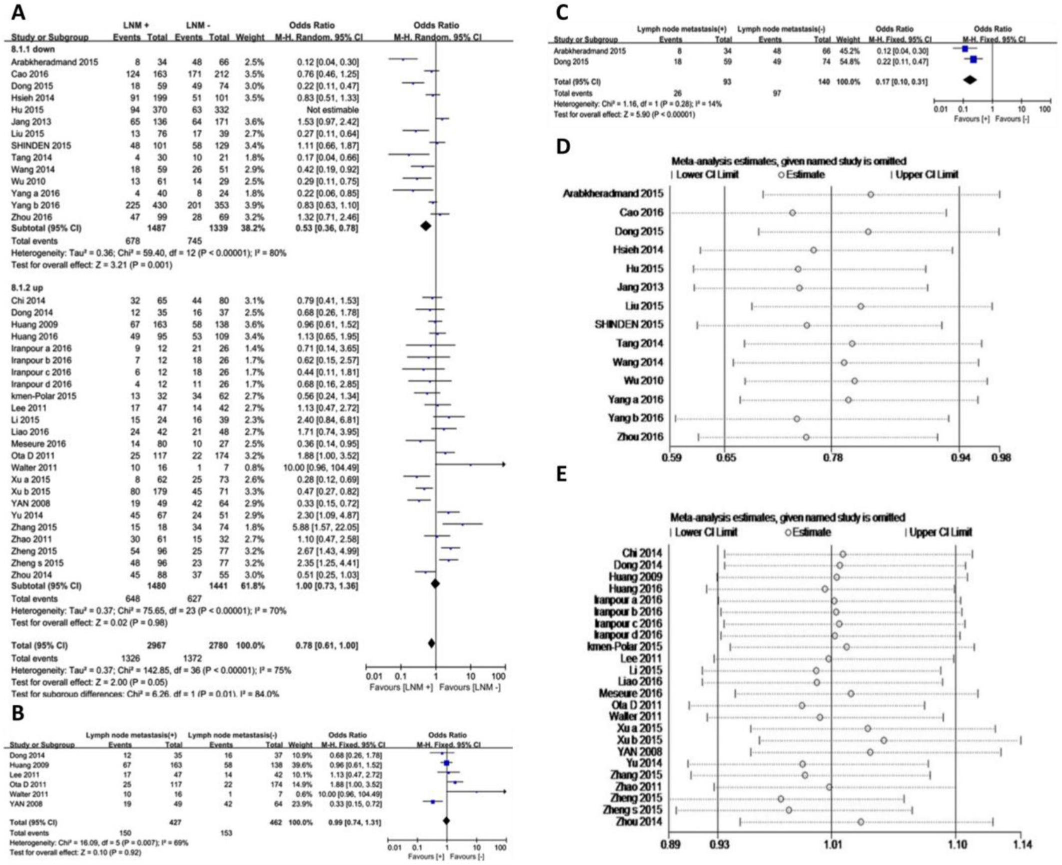

Supplementary Figure 11: Meta-analysis of lympho node metastasis. **(A)** Forest plots for the relation between ncRNAs and lympho node metastasis; **(B)** Forest plots for the relation between mir21 and lympho node metastasis; **(C)** Forest plots for the relation between mir124and lympho node metastasis; **(D)** Influence analysis for the relation between down-regulated ncRNAs and lympho node metastasis; **(E)** Influence analysis for the relation between up-regulated ncRNAs and lympho node metastasis.

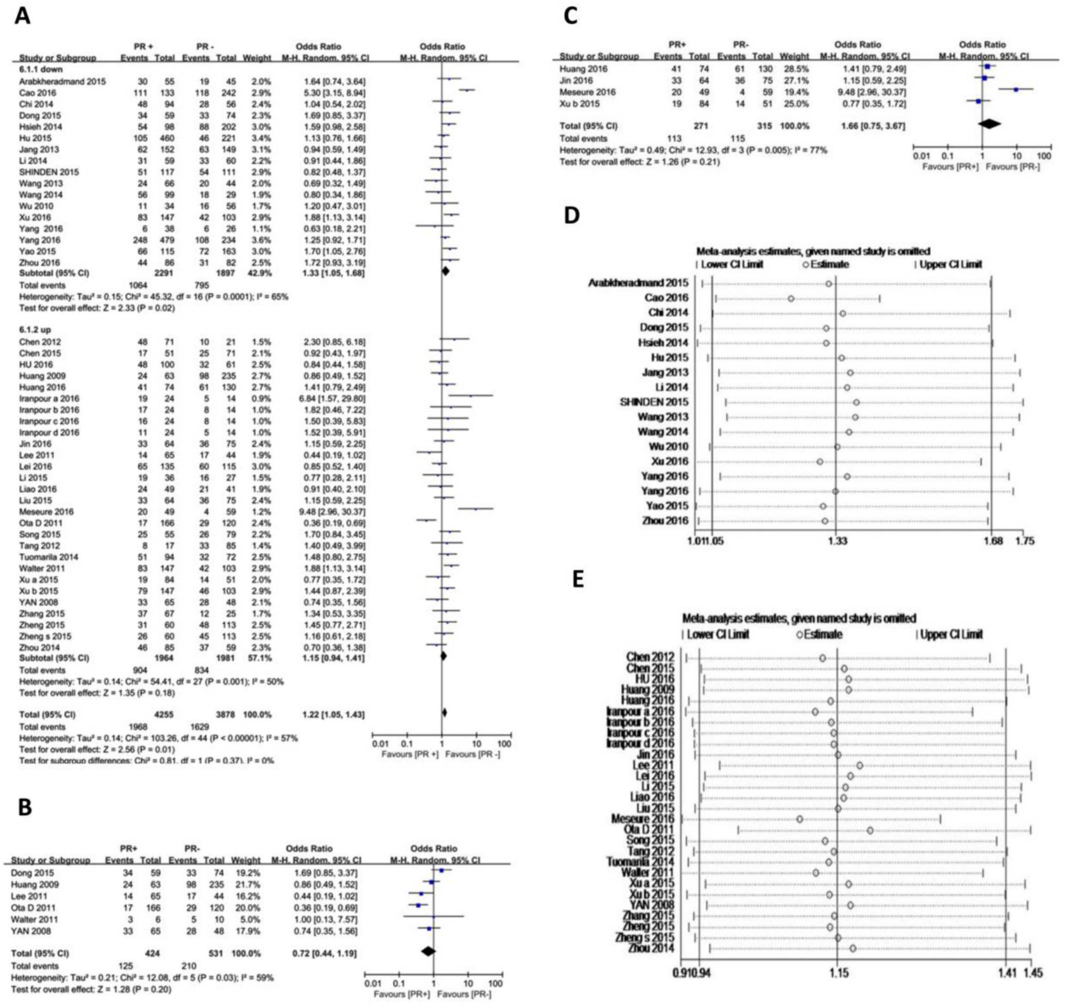

Supplementary Figure 12: Meta-analysis of PR. **(A)** Forest plots for the relation between ncRNAs and the expression of PR; **(B)** Forest plots for the relation between mir21 and the expression of PR; **(C)** Forest plots for the relation between MALAT1 and the expression of PR; **(D)** Influence analysis for the relation between down-regulated ncRNAs and the expression of PR; **(E)** Influence analysis for the relation between up-regulated ncRNAs and the expression of PR.

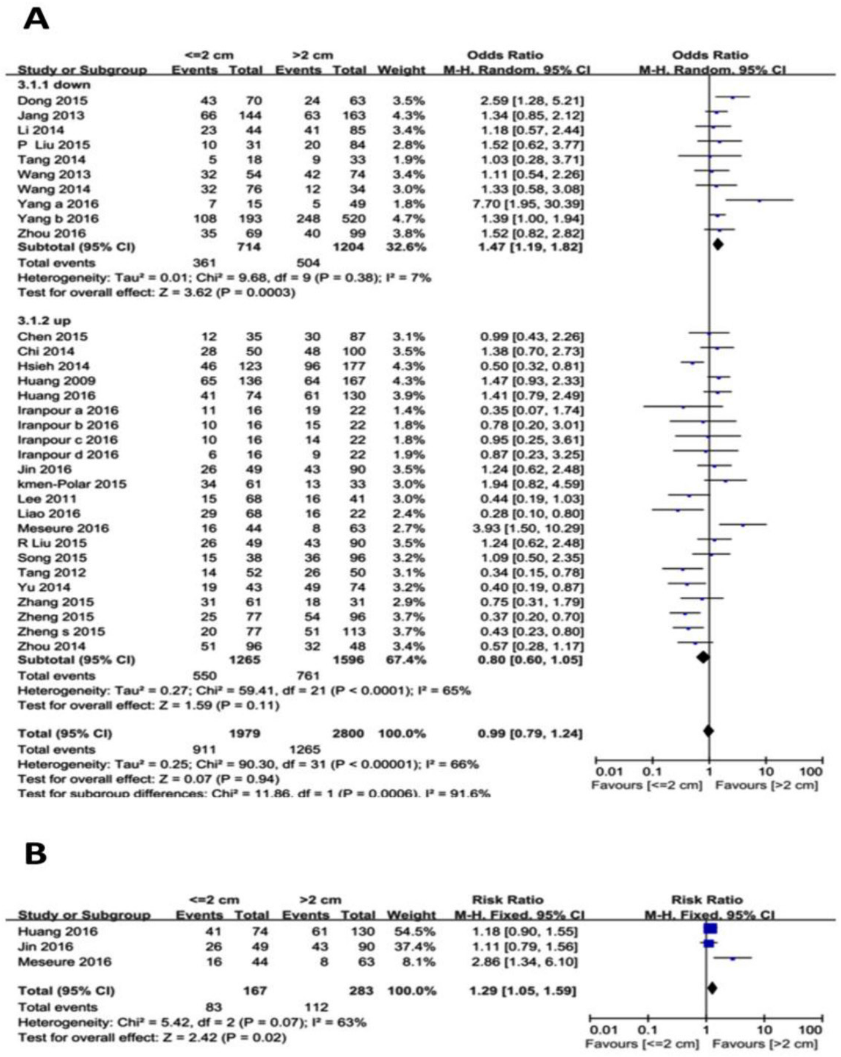

Supplementary Figure 13: Meta-analysis of tumor size. **(A)** Forest plots for the relation between ncRNAs and tumor size; **(B)** Forest plots for the relation between MALAT1 and tumor size.

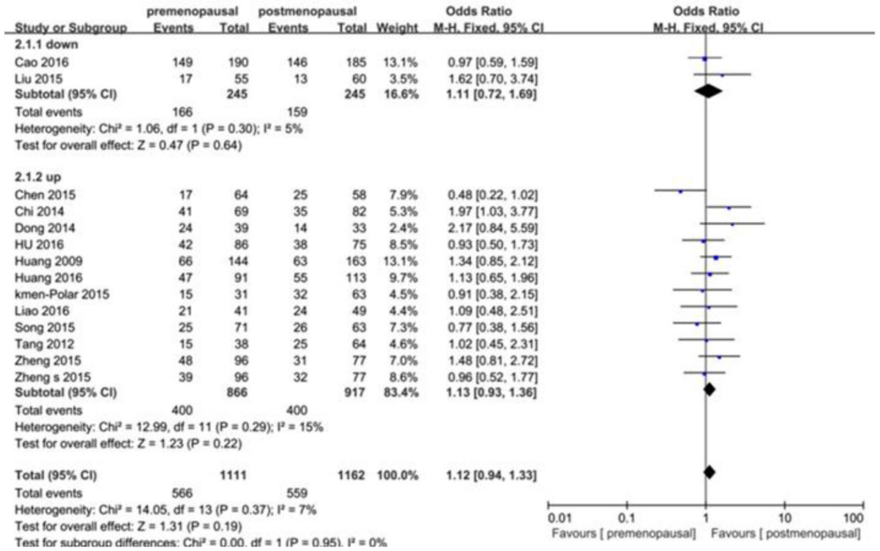

Supplementary Figure 14: Forest plots for the relation between ncRNAs and menopausal.

Supplementary Table. 1 Characteristics of studies included in the review of prognosis

See Supplementary file 1

Supplementary Table. 2 Characteristics of studies included in the review of Diagnosis

See Supplementary file 2

Supplementary Table. 3 Characteristics of studies included in the review of clinicopathological features

See Supplementary file 3
